# Neutrophils in respiratory viral infections

**DOI:** 10.1038/s41385-021-00397-4

**Published:** 2021-03-23

**Authors:** Cecilia Johansson, Freja C. M. Kirsebom

**Affiliations:** National Heart and Lung Institute, Imperial College London, London, UK

## Abstract

Viral respiratory infections are a common cause of severe disease, especially in infants, people who are immunocompromised, and in the elderly. Neutrophils, an important innate immune cell, infiltrate the lungs rapidly after an inflammatory insult. The most well-characterized effector mechanisms by which neutrophils contribute to host defense are largely extracellular and the involvement of neutrophils in protection from numerous bacterial and fungal infections is well established. However, the role of neutrophils in responses to viruses, which replicate intracellularly, has been less studied. It remains unclear whether and, by which underlying immunological mechanisms, neutrophils contribute to viral control or confer protection against an intracellular pathogen. Furthermore, neutrophils need to be tightly regulated to avoid bystander damage to host tissues. This is especially relevant in the lung where damage to delicate alveolar structures can compromise gas exchange with life-threatening consequences. It is inherently less clear how neutrophils can contribute to host immunity to viruses without causing immunopathology and/or exacerbating disease severity. In this review, we summarize and discuss the current understanding of how neutrophils in the lung direct immune responses to viruses, control viral replication and spread, and cause pathology during respiratory viral infections.

## Introduction

Neutrophils, the most abundant cell type in the blood in humans, are a fundamental component of the innate immune response. It is estimated that each day 1 billion neutrophils are produced per kilogram of body weight and that this can increase to 10 billion during an infection.^1^ At steady state, developing neutrophils reside in the bone marrow, while mature neutrophils are released into the circulation and rapidly recruited into affected tissues in response to infection or injury. Neutrophils are short-lived, although their precise life span is debated.^2,3^ As the most abundant and short-lived cell in the circulation, neutrophil turnover must be tightly regulated during both homeostasis and disease.

The role of neutrophils in host immunity is well described during bacterial and fungal infections. However, neutrophils are also detected in the lungs and/or bronchoalveolar lavage (BAL) of mice, rats, and humans after infection with respiratory viruses including human metapneumovirus (HMPV),^4–6^ human respiratory syncytial virus (HRSV; herein referred to as RSV),^7–11^ coronavirus,^12–17^ rhinovirus,^18–22^ measles,^23^ pneumonia virus of mice (PVM;^24^) mouse adenovirus type I,^25^ adenovirus 7,^26^ mouse cytomegalovirus (MCMV;^27^), and influenza A virus (IAV; reviewed in ref. ^28^). During such viral infections, where the pathogen replicates intracellularly, it is less clear whether neutrophil recruitment and activation benefit the host by contributing to host defense or whether their presence is a bystander effect of local inflammation and contributes to tissue damage and disease.^29,30^

## Neutrophils in human respiratory viral disease

Neutrophils are present in the lungs during acute respiratory distress syndrome, which can be induced by many different pathogens including many viruses (see table in ref. ^28^), as well as trauma and autoimmunity. Many studies suggest that neutrophil recruitment to the lungs is associated with disease severity during viral infections. For example, in infants with severe RSV-induced bronchiolitis, neutrophils can make up > 90% of the cellular composition of the BAL^10,11^ and therefore neutrophils have been implicated as drivers of disease pathogenesis.^10,11,31,32^ Also, in both rhinovirus and hMPV-infected children as well as in severe cases of influenza and SARS-CoV-2 infection, lung neutrophils and their markers have been observed to be elevated.^16,33–37^ Furthermore, whole-blood transcriptomic analyses have shown that genes related to neutrophil function and activation were among the overexpressed genes in infants hospitalized with RSV,^38^ in severely ill patients hospitalized during the 2009 IAV pandemic,^39^ and on the 1st day of hospitalization in patients that will require intensive care during SARS-CoV-2 infection.^40^ As neutrophil elevation is so commonly observed clinically during severe respiratory viral infections it is easy to speculate that their recruitment to the lung and further activation can enhance tissue pathology and contribute to disease. However, studying the causality of neutrophils in the human lung is challenging and therefore more detailed investigations into the function and role of neutrophils in viral respiratory disease have been performed in animal models. Many animal models of respiratory viral infections replicate the notable recruitment of neutrophils to the lung, and neutrophils are abundant in the airways and lungs of mice, calves, and ferrets during infections with RSV, IAV, coronaviruses, HMPV, and PMV.^4,7,9,17,24,41–44^ Here we discuss both the beneficial and detrimental effects of neutrophils during respiratory viral infections mostly from animal studies but also from human observations where data are available.

## Neutrophil recruitment

**Fig. 1:**
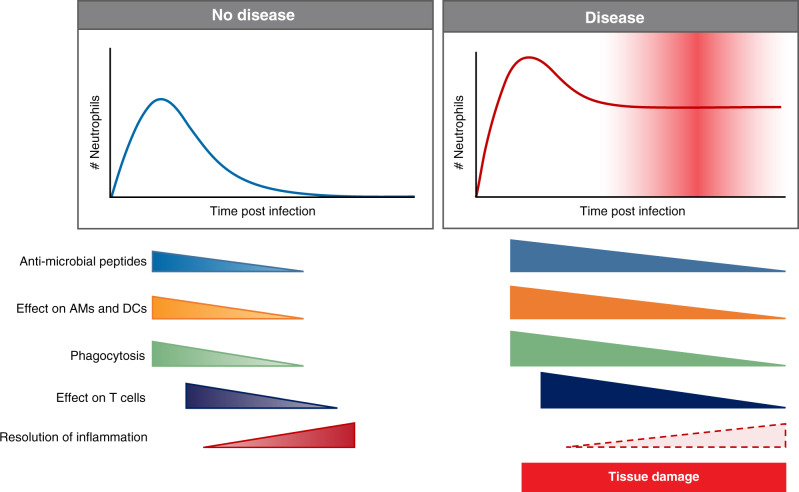
Neutrophils in disease. During a respiratory viral infection, neutrophils are recruited to and activated in the lung. In non-symptomatic or mild disease, neutrophil numbers peak early during infection and neutrophils exert their effector functions and aid in tissue repair and resolution of inflammation. In a severe infection, more neutrophils are recruited over a longer period. This results in more tissue damage and a delay or block in resolution of inflammation and tissue repair.

In the lungs of both mice and humans, a population of neutrophils resides in the pulmonary vasculature and perivascular space at steady state.^45,46^ It is thought that these neutrophils are retained in the lung actively by upregulation of the chemokine receptor CXCR4, which binds CXCL12, a ligand expressed by a subset of lung endothelial cells.^47^ The role of these resident lung neutrophils is not well understood, but it has been suggested that they localize in the pulmonary vasculature in order to be able to mount a rapid response to pulmonary pathogens.^45^ Nonetheless, a defining feature of neutrophils is their ability to infiltrate tissues early and rapidly during infections or sterile injury. For neutrophil recruitment to occur, pathogen-associated molecular patterns (PAMPs) and damage-associated molecular patterns (DAMPs) must trigger their respective receptors to initiate the production of pro-inflammatory mediators and neutrophil chemoattractants.^48,49^ Major neutrophil chemoattractants include interleukin-8 (IL-8; only in humans), CXCL1 (KC), CXCL2 (MIP2-α), CXCL5, complement component 5a (C5a), *N*-formylmethionine-leucyl-phenylalanine (fMLP), platelet activating factor, and leukotriene B4 (LTB4).^48,50–52^ During inflammation, neutrophil transmigration to the affected tissue occurs in a step-wise process known as the leukocyte adhesion cascade.^3,53^ Many neutrophil chemoattractants are produced in the lungs and airways during viral infections, for example, CXCL1, CXCL2 and IL-17,^7,17,25,28,50,54–57^ resulting in neutrophil infiltration into the lungs of mice and ferrets.^7,8,41,58–60^ During RSV infection in mice this infiltration to the lungs is transient as neutrophils peak at 18 h postinfection (p.i.) and are almost absent by 36 h p.i.^7–9,61^ This is different during severe mouse IAV infection, in which neutrophils chemoattractants such as CXCL1 and neutrophils remain in the lungs for a longer time and are still detectable at day 12 p.i., especially when a highly pathogenic IAV strain is used.^62–66^ This would suggest that a sustained neutrophil recruitment and/or presence in the lungs is associated with more severe disease (Fig. 1).

PAMPs from the virus, or from the infection process, are recognized by pattern recognition receptors (PRRs), through which signaling is crucial to initiate the inflammatory response.^67–71^ During RSV infection, neutrophil recruitment to the lung is dependent on MyD88^72,73^ or MyD88/TRIF^7,74^ signaling, which occurs downstream of a class of PRRs known as the toll-like receptors (TLRs), as well as cytokine receptors of the IL-1R family. *Mavs*^−/−^ mice (unable to signal via cytosolic PRRs of the RIG-I-like family, which detect PAMPs predominantly associated with RNA viruses) do recruit neutrophils to the lung early after RSV infection, albeit fewer than wild-type mice.^7^ MyD88 signaling is also essential for neutrophil recruitment to the lung during IAV infection^64,75,76^ and during infection with mouse-adapted SARS-CoV.^77^ The role of TLR3 in recruitment of neutrophils to the lung during IAV infection is somewhat controversial as IAV-infected *Tlr3*^−/−^ mice have shown reduced,^64^ increased,^78^ or similar^76^ neutrophil infiltration compared to wild-type mice. As different viral strains and different time points were used this will be important to study in detail in future studies as the magnitude and timing of the neutrophil responses might be dependent on which PRRs that are used for induction of the neutrophil attractants. Viral proteins can also contribute to the regulation of neutrophil recruitment. For example, during HMPV infection the attachment glycoprotein is involved in neutrophil infiltration.^79^

The key cell types in which PRR signaling takes place to induce production of neutrophil chemoattractants, or cytokines that can induce chemoattractants, appear to vary between pathogens. Signaling in non-hematopoietic cells was shown to be required for neutrophil recruitment during IAV infection.^64^ Furthermore, during RSV infection non-epithelial cells (ATII cells), non-endothelial, lung stromal cells were shown to be important for *Cxcl1* induction in a MyD88/TRIF signaling dependent manner.^7^ The precise source of neutrophil chemoattractants during viral infection is an important research avenue for future directed therapies to reduce neutrophilic inflammation.

During RSV infection of both mouse and man, there are two temporally distinct waves of neutrophil-attracting chemokines. The first wave of chemokines is produced early after infection and then a later induction occurs concurrently with the peak of disease symptoms.^56,80^ Interestingly, neutrophil infiltration also occurs in two distinct waves during IAV infection of ferrets.^60^ External factors that regulate neutrophil-attracting chemokines are not yet well understood but it is not thought that the circadian rhythm influences neutrophil infiltration to the lung during IAV infection.^81^ In RSV-infected infants, neutrophils are the predominant cell type in the BAL.^10^ However, this is at a later stage of infection when children are admitted to the hospital with symptoms. When studied early after RSV infection in the neonatal mouse model relatively few neutrophils infiltrate the lungs.^82,83^ Furthermore, *Cxcl1* is not induced in lungs of neonatal mice after RSV infection.^84^ However, *Cxcl1*, CXCL2, and neutrophils are detected in the lung tissue and airways of neonatal mice after IAV infection.^84,85^ If the relatively reduced neutrophil recruitment to the lower airways in the early stages of RSV infection of neonatal mice is also a phenomena of RSV infection in infants is not yet clear. Sampling infants prior to symptoms is extremely challenging and therefore rarely done except for perhaps in birth cohort studies, which can include longitudinal nasal sampling, virus detection, and symptom scoring. Therefore, little is known about the early stages of disease caused by respiratory viral infection in children so far and more studies are needed. Overall, neutrophils commonly infiltrate the lungs of both humans and animals during all respiratory viral infections but interestingly both the magnitude and timing of neutrophil infiltration is dependent on the type of infection studied.

## Neutrophil priming and activation

**Fig. 2:**
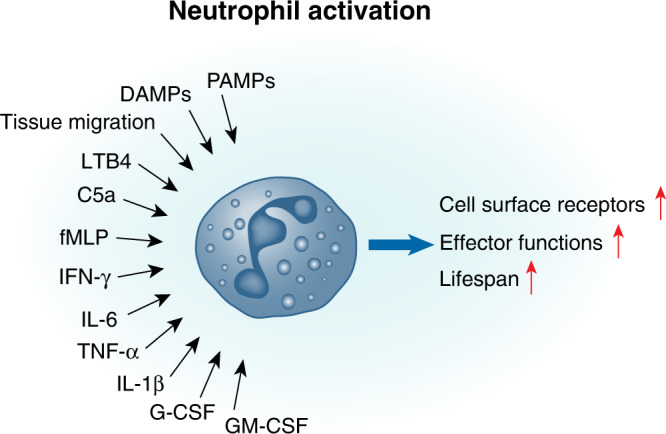
Multiple ways to activate a neutrophil. Neutrophils receive many different signals from the inflammatory environment that can lead to cell activation and elicit effector functions. These activating signals include PAMPs, DAMPs, the process of migration, neutrophil chemoattractants, and cytokines.

Activation is essential for neutrophils to exert their full antimicrobial functions and contribute to host defense (Fig. 2). Historically, neutrophils were considered as unsophisticated responder cells, yet it is increasingly evident that the role of neutrophils in inflammation is more complex than has previously been appreciated. Neutrophils can respond differentially to harmful stimuli,^86,87^ interact with other arms of the immune response,^88^ and can also have roles in wound healing and resolution of inflammation.^89^ Neutrophil activation is a process that occurs over time, starting already during recruitment.^90^ However, in order to become fully activated to degranulate and be capable of undergoing oxidative burst, neutrophils must undergo a further series of priming steps (Fig. 2). As neutrophil activation can be highly destructive and cause local damage to host tissues, driving immunopathology,^91^ this multistep priming process for activation acts as a mechanism to safeguard against these potentially damaging effector functions.

### Factors that activate neutrophils

Once in the tissue, an inflammatory tissue micro-environment containing host-derived compounds such as TNF-α, IL-1β, IFN-γ, and GM-CSF can drive neutrophil activation as well as amplify neutrophil recruitment to the site of infection^7,92–95^ (Fig. 2). Compounds released during tissue damage can also trigger activation; heme, released abundantly from damaged cells such as red blood cells, was shown in vitro to potently trigger oxidative burst in human neutrophils in a dose dependent manner.^92^ In addition, neutrophils express a broad repertoire of PRRs, including TLRs (TLR1/2, TLR2/6, TLR4, TLR5, TLR7–TLR9), RIG-I-like receptors (RIG-I, MDA-5), C-type lectin receptors, and NOD-like receptors, all of which can directly bind PAMPs.^96^ Neutrophil chemoattractants such as LTB4, C5a, and fMLP can also activate neutrophils.^90^ Binding of these factors to their respective receptors (often G-protein-coupled receptors) triggers a downstream signaling cascade often via the MAPK/ERK pathway,^95,97^ which then induces neutrophil effector functions such as oxidative burst and degranulation (see details below). The concentration of host chemokines can also impact the effect these mediators have on neutrophil activation status; for example, IL-8 induces shedding of L-selectin and upregulation of certain integrins at low concentrations, while at higher concentrations it can induce the oxidative burst.^53,90^ Binding of IL-1β can directly induce reactive oxygen species (ROS) production in human neutrophils in a MAPK-dependent manner, while stimulation with GM-CSF can activate neutrophils in an ERK-dependent manner.^98^ Notably, stimulation of human neutrophils with both IL-1β and GM-CSF has an additive effect, resulting in activation of both MAPK and ERK pathways, and demonstrating how activation can be enhanced by the presence of multiple stimuli.^98^ PAMPs can also act synergistically on neutrophils; for example, lipopolysaccharide (LPS) can induce assembly of the cellular machinery required for oxidative burst on the membrane of neutrophils, while recognition of fMLP provides the final signal to drive the production of ROS.^99,100^

Multiple inflammatory signals have been demonstrated to increase neutrophil life span including IFN-γ, GM-CSF, G-CSF, IL-6, and PAMPs such as LPS (Fig. 2).^101–103^ It has also been suggested that RSV-induced neutrophil activation delays apoptosis in vitro,^104^ but whether this would be beneficial or detrimental to disease outcome during infection in vivo remains unclear. In vitro studies of virus-induced activation of neutrophils should be carefully considered as the inflammatory mediators produced by the cell line in which the virus is propagated could result in neutrophil activation. For example, a study using RSV “washed” of pro-inflammatory mediators produced by epithelial cells shows that this RSV preparation stimulates neutrophils in vitro significantly less compared to neutrophils stimulated with “unwashed” RSV,^105^ suggesting that RSV particles on their own do not stimulate neutrophils. Overall, the activation of neutrophils will be very dependent on the inflammatory environment they encounter once entering the lungs (Fig. 2) and it is possible that a more severe infection results in a different, larger, and prolonged presence of neutrophil activation signals that then drives an excessive neutrophil effector response (Fig. 1) contributing to disease severity.

### Cell surface receptor upregulation

Several cell surface receptors change on neutrophils after recruitment and activation. For example, CD64 (high affinity FcγRI), CD11b, and CD69 can be upregulated and CD62L and CD182 downregulated on neutrophils after activation.^59,102,106–108^ CD69 has, in mice, been demonstrated in mice to be upregulated on BAL neutrophils specifically in response to IAV,^59^ however, this was not observed during RSV infection.^7^ Furthermore, on blood neutrophils from IAV-infected humans, CD11b was upregulated,^109^ while CD64 expression was upregulated in one study^110^ and downregulated in another study.^109^ Also, during rhinovirus infection of chronic obstructive pulmondary disease (COPD) patients, sputum neutrophils upregulated CD11b, CD63, and CD66.^21^ In mice, CD64 was the only marker specifically upregulated on lung neutrophils in response to RSV infection.^7^ Triggering of CD64 drives an intracellular signaling cascade, which has been suggested to drive actin polymerization and facilitate phagocytosis.^111^ Increased phagocytosis by neutrophils could have a role in clearing up debris from dying cells in the lung, as has been reported in other inflammatory contexts.^112^ Furthermore, Fc receptors such as CD64 can bind opsonized pathogens and immune complexes.^113^ Therefore, it is possible that later during a primary infection or during a reinfection when virus-specific IgG are present, activated neutrophils can increase phagocytosis of IgG-bound viral particles and have a more pronounced role in viral clearance. This has so far not been studied in detail and will be an important avenue for future studies.

The magnitude and the combination of specific mediators in the inflammatory environment will drive neutrophil activation and determine the extent and type of their effector programs initiated (see below). Overall, the data so far suggest that neutrophils are differentially activated depending on the specific respiratory virus in question and support recent literature that neutrophils can tailor their response during activation to specific pathogens by reacting to a certain mix of activating signals (Fig. 2).^87^

## Neutrophil effector functions in the antiviral response

**Fig. 3:**
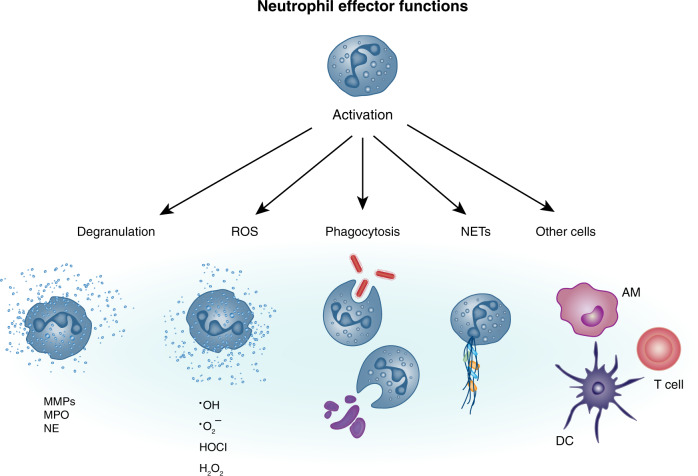
Neutrophils exhibit a plethora of effector functions. Neutrophil activation can trigger degranulation whereby neutrophils secrete mediators and proteolytic enzymes such as MMPs, MPO, and NE, which are prestored in cytoplasmic granules. Neutrophils can also mediate pathogen clearance by producing ROS, which can either occur intracellularly in the phagolysosome to kill internalized microbes or extracellularly, to combat larger pathogens. Phagocytosis of pathogens can limit microbial spread, while phagocytosis of cellular debris and apoptotic material can contribute to the resolution of inflammation. Neutrophils can also restrict microbial spread by secreting their chromatin as NETs, trapping pathogens. Finally, it is increasingly appreciated that neutrophils can have direct and indirect interactions with other cells such as alveolar macrophages (AM), dendritic cells (DC), and T cells, which can contribute to both innate and adaptive immunity.

Activated neutrophils have many different functions (Fig. 3). In recent years, experimental evidence has suggested that there may be various subtypes of neutrophils with different roles in infection, cancer, and autoimmunity.^114^ These subtypes have been defined based on size (N1 and N2 neutrophils)^115,116^ or density (low-density neutrophils (LDNs) and high-density neutrophils).^117,118^ Furthermore, both immunosuppressive and pro-inflammatory LDNs have been described.^117,118^ Immunosuppressive LDNs have been found to suppress T-cell proliferation and IFN-γ production and may have a more immature phenotype in some situations.^117^ However, it is not yet known whether these subtypes have differing functions in tissue damage or host defense against respiratory pathogens. It is also unclear if the phenotypic differences represent true developmentally distinct subtypes or whether these differences can be attributed to differences in the priming, maturation and/or activation status of neutrophils. In addition, data on human lung neutrophils are very limited but potential differences between mouse and human neutrophils in the context of subtypes need to be further investigated.

### Degranulation

Neutrophil granules contain > 1200 unique proteins prestored in membrane-bound vesicles in the cytoplasm.^119^ These include proteolytic enzymes, antimicrobial proteins, components of the NADPH oxidase as well as membrane-bound receptors for endothelial adhesion molecules, extracellular matrix proteins, and soluble mediators of inflammation.^120^ Degranulation, the secretion of neutrophil granules, is a critical effector function of neutrophils initiated early during neutrophil recruitment. Neutrophils contain three types of granules, primary (azurophil), secondary (specific), tertiary (gelatinase) granules, and also secretory vesicles.^121,122^ Degranulation occurs in a sequential manner; first to be released are the secretory vesicles, while azurophilic granules are last, requiring the most potent activation signals for their release.^121^ Neutrophil granule contents have widespread effector functions. Gelatinases and collagenases such as MMP-8 and MMP-9 (stored in secondary and tertiary granules) aid neutrophils in their migration through the extracellular matrix.^64,123^ As they degrade extracellular matrix, this results in release of proline–glycine–prolines, which act as further neutrophil chemoattractants.^124^ MMP-9 has also been shown to be important for viral clearance during RSV infection of mice.^125^ Other proteins are thought to be directly antimicrobial, such as neutrophil elastase (NE) and myeloperoxidase (MPO) (stored in primary granules), which can either act proteolytically or by catalyzing ROS production.^126^ NE has been detected in the serum and airways of infants hospitalized with RSV infection.^127,128^ The detection of NE suggests that neutrophils recruited to the lung during RSV infection are fully activated. However, the relationship between this and the putative role of neutrophils in disease protection versus pathogenesis is not clear. Antimicrobial peptides including the cathelicidins (e.g., LL-37) and the alpha-defensins are stored in neutrophil granules.^90,121^ Cathelicidins are activated upon cleavage by proteinase 3^129^ and can be directly antimicrobial, or contribute to host defense by inducing or modulating chemokine and cytokine production.^130^ Alpha-defensins have antimicrobial activity against a wide range of bacteria, fungi, and enveloped viruses; the mechanism of action is thought to be via disruption of the plasma membrane by pore formation or by covering the pathogen.^131^ For example, human cathelicidin LL-37 (produced by both neutrophils and epithelial cells) can inhibit IAV and RSV infection^132–134^ and human neutrophil peptides, released from neutrophils, can inhibit IAV infectivity by increasing IAV uptake by neutrophils via a mechanism probably involving viral aggregation.^135,136^ Interestingly, airway secretions from RSV-infected children contain more neutrophil-mediated antibacterial activity compared to RSV-negative controls.^137^ The extent of neutrophil degranulation must be carefully balanced against the potential harm caused by the pathogen as the dysregulated release of proteolytic enzymes by neutrophils can degrade the extracellular matrix, contributing to immunopathology^91^ (see more below).

### ROS

Oxidative burst via the production of ROS is a powerful tool to eliminate pathogens. In neutrophils this is largely mediated by the NADPH oxidase enzyme complex (reviewed elsewhere^138,139^) ROS are thought to be harmful to pathogens in multiple ways; directly by causing damage to the pathogen as well as indirectly by inducing autophagy, inhibiting mTOR kinase to trigger an antiviral response, promoting NETosis (see below) and by promoting cell death of infected cells that act as pathogen reservoirs.^140^ Using different physical forms of the fungal pathogen *Candida albicans*, it was recently demonstrated that ROS localization can act as a mechanism for neutrophils to sense microbe size and that this influences the ensuing neutrophil response.^86,87^ Spores of “small” *C*. *albicans* induced intracellular ROS production in phagosomes, while “large” *C*. *albicans* hyphae induced extracellular ROS. The induction of intracellular ROS in response to “small” pathogens suppressed IL-1β production and restricted the recruitment of more neutrophils to the site of infection, while extracellular ROS in response to a “large” pathogen had the opposite effect on IL-1β production and neutrophil recruitment.^86,87^ These findings suggest that the induction of ROS, in addition to the well-known role in pathogen removal, also has an important role in directing the ensuing neutrophil response. Oxidative burst has been reported during IAV, PVM, and RSV infection in mice^24,105,141^ and RSV have also been shown to cause oxidative stress in epithelial cells.^142^ It is therefore interesting to speculate that ROS levels during virus infection could be limiting the neutrophil response.

### Phagocytosis

As professional phagocytes, neutrophils contribute to host defense by clearing up pathogens, dead cells, and other debris during inflammation.^3,143^ Pathogens opsonized with complement and antibody can be engulfed following interactions with their respective receptors on neutrophils, the complement receptors and FcγRs, such as CD64^113^ (discussed above). For example, surfactant protein D can opsonize RSV and IAV for neutrophil phagocytosis that then results in ROS production.^144,145^ Following phagocytosis, preformed granules in the cytoplasm of the neutrophil containing hydrolytic enzymes and NADPH oxidase will fuse with the phagosome.^143^ The pathogen can either be destroyed enzymatically or by ROS in the mature phagosome. Also, neutrophils can phagocytose infected, apoptotic cells, which are important for resolution of inflammation^146^ (see more below).

### Neutrophil extracellular traps (NETs)

A more recently described effector function of neutrophils is their ability to secrete NETs^147–151^ (Fig. 3). NETs are large extracellular structures of modified, decondensed chromatin and DNA decorated with the protein contents of neutrophil granules.^150^ NETs are induced during infections with bacteria, fungi, parasites, and viruses.^150^ Multiple factors can trigger neutrophils to secrete NETs and several molecular pathways regulate their secretion.^150^ The main way NETs are induced is via a form of cell death termed NETosis, which is distinct from apoptosis and necrosis, and dependent on ROS production by NADPH oxidase.^152^ Alternatively, NETs can be induced in a manner that does not kill the neutrophil in a process termed non-lytic NETosis.^153,154^ This is independent of ROS production and occurs by exocytosis of vesicles filled with nuclear DNA.^154^ Interestingly, this leaves behind both neutrophil cytoplasts with both diffuse, intracellular cytoplasmic DNA and completely anucleated cytoplasts, neutrophil “ghosts”^155^ (more about these below). The induction of NETs is thought to be especially beneficial in host defense against larger pathogens, which cannot be removed by phagocytosis.^86,87,150^ However, during viral infections it is possible that damaged cells trigger NETosis instead of the virus itself, or that different triggers in addition to pathogen size regulate whether neutrophils undergo NETosis. Patients with chronic granulomatous disease have a defect in expressing fully functional NADPH oxidase and therefore have impaired ROS and NET production.^156^ These patients develop frequent and/or severe bacterial and fungal infections but are not particularly susceptible to viral pathogens.^29,156^ However, NETs in both lung tissue and serum of severe COVID-19 patients have been detected^14,35,157,158^ and patients with severe H1N1 and H7N9 IAV infection showed high concentrations of NETs in their serum.^159^ In addition, in mouse in vivo studies, NETs were detected in areas of tissue injury in the lung during infection with a mouse-adapted IAV strain (PR8) or Sendai virus^160–163^ as well as during rhinovirus infection in an allergic asthma mouse model.^164^ As NETs can trap pathogens, these structures could be beneficial for the host. Indeed, histones have been shown to neutralize H3N2 and H1N1 IAV^165^ and NETs have been suggested to capture RSV particles in vitro.^43^ It is also possible that NETs are used to plug holes from dying epithelial cells in the barrier but this is still controversial and yet to be formally proven. However, it has been shown that peptidylarginine deiminase 4-mediated NET formation is not required for the host response and survival during IAV infection^166^ but several studies suggest that excessive neutrophil influx during IAV infection results in the release of toxic NETs and granule enzymes, which are associated with pulmonary pathology.^160,167,168^

During PVM infection, very few NETs are detected,^24^ but there is evidence that RSV infection can trigger neutrophils to excrete NETs.^24,42,43,169–171^ Neutrophils isolated from the airways of patients with RSV-induced bronchiolitis and cultured ex vivo expelled strands of DNA, as detected by staining with the DNA stains Hoechst and SYTOX.^170^ Furthermore, formation of NETs in lung tissue sections from RSV-infected calves was detected by staining for citrullinated histone H3, a more specific way to detect the presence of NETs than DNA stains.^24,43^ In mice, the two major studies that have tried to visualize NETs in vivo during RSV infection are limited by the use of a DNA stain, which detects both NETs and extracellular DNA.^169,171^ Therefore, it cannot be excluded that these studies are also detecting cell death in the lung in response to RSV infection. Nonetheless, these studies showed that RSV could induce NET structures in the lung and, more specifically, RSV F protein alone was possibly sufficient to induce MPO-coated NETs in vivo.^169,172^ The role of NETs during viral infection is debated and whether NETs can benefit the host in defense from viruses, which are largely intracellular, or if NETs contribute to tissue damage is still being evaluated.^30^

### Activation and regulation of other cells

Neutrophils are thought to be a source of cytokines and chemokines during infections^173^ and they can modulate the function of other immune cells^174^ (Fig. 3). For example, lung neutrophils constitutively express pro-IL-1β.^175^ Recently it has been shown that neutrophils regulate the production of IL-1β by alveolar macrophages (AMs) during infection with IAV.^58^ However, during RSV infection, no differences in IFN-α, IL-1β, or IL-6 levels in BAL were detected after neutrophil depletion,^176^ suggesting neutrophils do not modulate the cytokine production by AMs during RSV infection. Furthermore, TNF-α levels were lower after neutrophil depletion during RSV infection in one study^177^ but not in another study.^176^ The reasons for the disparity between these studies may be due to the mouse or viral strains used or the time point studied. Overall, cytokine/chemokine responses during neutrophil depletion are not massively altered^58,176,177^ so it is important to carefully establish which cytokines/chemokines neutrophils produce during specific infections and their contribution to the overall inflammatory environment. Furthermore, neutrophils have been shown to inhibit lung inflammation by reducing the accumulation of γδ T cells during HMPV infection^4^ and they could also inhibit inflammation via NE cleaving TLRs on macrophages and co-receptors on T cells.^174^ It is very likely that neutrophils and/or the mediators they secrete regulate the activation of other cell types in the lung and future studies will be required to further uncover the impact of this in the overall antiviral response.

## Viral replication and clearance

Whether neutrophils can have direct antiviral effects during respiratory viral infections is debated and has been explored using animal models. Interestingly, after antibody mediated neutrophil depletion in mice there is overall very limited effect, if any, on the viral load during RSV, IAV, PVM, or HMPV infection (Table 1). However, in a study where neutrophils were depleted in rats during rat coronavirus infection, an increase in viral load was found.^13^ Also, in MCMV infection neutrophil depletion resulted in increased viral load in the lung and this study suggested that neutrophils use TRAIL for viral control.^27^

**Table 1:** The effect of neutrophil depletion in mice on viral load, weight loss and lung pathology during multiple respiratory viral infections.

Virus	Viral load	Weight loss/clinical score	Pathology and cell infiltration	Viral strain	Species and strain	Refs.
IAV	–	–	?	X31	Mouse; BALB/c	^58^
IAV	↑	↑	↑	X31	Mouse; C57BL/6	^44,65^
IAV	↑	↑	?	PR8	Mouse; C57BL/6	^65^
IAV	–	–	–	PR8	Mouse; BALB/c	^160^
IAV	–	↑	?	PR8	Mouse; C57BL/6	^66^
IAV	–	↑	?	PR8	Aged mice; C57BL/6	^66^
PVM	–	–	–	J3666	Mouse; BALB/c	^24^
PVM	–	–	–	J3666	Mouse; C57BL/6	^24^
RSV	–	?	?	2-20	Mouse; BALB/c	^177^
RSV	–	–	?	A2	Mouse; C57BL/6	^176^
HMPV	–	↑	↑	CAN97-83	Mouse; BALB/c	^4^
MCMV	↑	↑	?	Smith strain	Mouse; C57BL6	^27^
rCoV	↑	↑	↓	Sialodacryoadenitis virus	Rat; Fisher 344	^13^

– no difference; ↑increase; ? not evaluated.

It has been suggested that the massive neutrophil recruitment to the lung early during infection might provide cellular sites for viral replication.^178^ One study did indeed detect RSV mRNA transcripts in neutrophils isolated from infants with RSV-induced bronchiolitis.^178^ However, it remains to be established whether any viruses have the ability to replicate productively in neutrophils in vivo. Overall, the findings that neutrophil depletion early during infection does not generally alter lung viral loads in vivo (Table 1) suggest that they are not important host cells for viral replication nor are they major players in the control of viral replication or spread of most respiratory viruses.

## Links to adaptive immunity and disease severity

### Neutrophils influencing T and B cells

In recent years, it has become increasingly well appreciated that neutrophils can play a role in directing several aspects of adaptive immunity.^179,180^ It has been suggested that neutrophils can interact directly with dendritic cells (DCs) to enhance their antigen presentation ability,^181,182^ as well as indirectly, by secreting granule contents that then act on DCs^183,184^ (Fig. 3). In some contexts, neutrophils can act as antigen presenting cells (APCs) themselves^185–187^ and monocytes recruited to the trachea during IAV infection can engulf apoptotic neutrophils and serve as important APCs for restimulating T cells in the tissue.^188^ Furthermore, during IAV infection, the early influx of lung neutrophils has been shown to influence the later recruitment of antiviral CD8^+^ T cells,^189,190^ for example, by leaving behind trails of the T cell-attracting chemokine CXCL12.^189^ Conversely, it has also been suggested that CD11b^+^ neutrophils suppress T cells and limit T cell-mediated lung pathology during IAV infection.^191^ However, although neutrophil depletion during IAV infection disrupted CD8^+^ T cell recruitment to the lungs^192^ and trachea^188^ and also the functionality of these cells,^188^ it did not affect the formation of a functional resident T cell memory population nor did it affect susceptibility to lethal heterosubtypic IAV challenge.^192^ In contrast, during RSV infection, neutrophil depletion did not change the CD4^+^, CD8^+^, or RSV-specific CD8^+^ primary (day 8 p.i.) or memory T cells responses observed in the lung.^176^ Therefore, it is possible that neutrophils may have dual roles in balancing T cell immunity versus T cell-driven tissue damage during respiratory viral infections.

Neutrophils have also been suggested to contribute to the enhancement of B cell responses in some inflammatory contexts, thereby contributing to the development of antibody responses.^180,193,194^ This is in part due to the production of the cytokines BAFF and APRIL, which are regulators of B cell survival and activation.^195^ However, whether this contributes to the functionality of the antibody response is not clear. During IAV infection, it was shown that human neutrophils do not bind or internalize IAV immune complexes.^196^ However, in a mouse study, passively transferred serum from IAV-infected mice protected against infection and this protective effect was blocked by depleting recipient mice with an anti-GR-1 antibody, which is partially specific for neutrophils.^197^ Together, these data suggest that neutrophils can impact the induction of adaptive immune responses and/or the effect of such responses but the mechanisms used might differ in different viral infections and in primary versus memory responses to a specific virus infection.

### Neutrophils in tissue damage and disease severity

Neutrophil recruitment alone does not appear enough to cause substantial tissue damage as artificial attraction of neutrophils into the lung using recombinant CXCL1 does not increase weight loss during RSV infection,^176^ a key measure of pathology and disease severity in this model.^198,199^ Therefore, neutrophils have to receive further signals from the virus-induced lung inflammatory environment^28,69,200^ to become fully activated and perform their effector functions.^7^ Many of the neutrophil effector mediators can, at high concentrations, damage tissues. For example, NE digests the extracellular matrix^201^ but can also drive mucus production, which can aid in pathogen clearance but also contribute to disease as mucus plugs can block the airways.^202^ NET release together with mucus production can also be detrimental by increasing tissue damage and impairing lung function.^43,172^ Neutrophil depletion studies during IAV infection provide conflicting evidence on whether the net effect of neutrophil recruitment and activation is to drive disease or to contribute to host defense.^44,58,65,66,160,161,189,190^ This is in part due to virus strain-specific differences as the number of neutrophils infiltrating the lungs during IAV infection is highly strain dependent.^65,175,203^ The mouse-adapted PR8 IAV strain induces more severe disease, in part thought to be mediated by neutrophils, while infection of mice with other IAV strains, causing milder disease, does not appear to induce neutrophil-driven pathology to the same extent (Fig. 1).^65^ Interestingly, age also seems to influence disease severity during respiratory infections as excessive neutrophil levels were found during SARS-CoV-2 infection^204^ and during IAV infection of aged mice.^66^

### Neutrophils and resolution of inflammation

Neutrophils can aid in disease resolution and wound healing (as reviewed extensively in^205,206^). They can also contribute to resolution of inflammation by clearing up virus-infected cells by phagocytosis.^146^ In addition, apoptotic neutrophils taken up by macrophages via efferocytosis signal to macrophages to switch to a more anti-inflammatory phenotype.^205,207^ Neutrophils can also contribute to epithelial cell proliferation, important for keeping the barrier intact,^112^ and they can secrete pro-resolution products such as annexin A1.^205^ However, during respiratory viral infections, the contribution of these possible functions to disease resolution is not well understood, and it has been shown that human neutrophils can increase the epithelial cell damage during in vitro RSV infection.^208^ As neutrophils have been shown to deplete local O_2_, which increases resolution of acute colonic inflammation,^209^ it is possible that this is also the case in the airways. Although it has also been shown that hypoxia augments neutrophil degranulation and killing of airway epithelial cells^210^ and therefore could potentially cause more lung tissue damage. An interesting aspect of neutrophil depletion experiments during respiratory viral infections in mice is that several of the models showed increased weight loss or clinical scores when neutrophils were lacking in the early phases of infection (Table 1). This could suggest that neutrophils have an important role in the resolution phase as resolution of any inflammatory responses in the lungs is important for restoring steady state. Future detailed studies will inform on how neutrophils contribute to these processes and if and when they can potentially be manipulated to increase this function.

## Neutrophils in co-infections and viral exacerbations of asthma

### The role of neutrophils in co-infections

Co-infections with several viruses can occur, but the neutrophilic response in nasopharyngeal aspirate samples from children is not altered in the presence of several simultaneous virus infections.^33^ A secondary bacterial infection is a common feature after severe respiratory viral infections, and is often the cause of death.^211–213^ For example, the 1918 “Spanish flu” pandemic is thought to have reached the devastating death toll due to secondary bacterial infections.^214^ Several studies have shown a defect in subsequent neutrophil function after a respiratory viral infection. For example, IAV-induced neutrophil dysfunction contributed to increased susceptibility to a secondary *Streptococcus pneumoniae* infection^215^ and neutrophil depletion during *S*. *pneumoniae/*IAV co-infection increased mortality.^216^ Also, the production of neutrophil chemokines (CXCL1 and CXCL2) was impaired during *S*. *pneumoniae* infection of mice which had had prior IAV or HMPV infection.^217,218^ Interestingly, neutrophils in *S*. *pneumoniae* or *S*. *pneumoniae/*IAV co-infected mice did not show a functional difference in ROS, NET, or cytokine production,^216^ while human neutrophils simultaneously incubated with IAV and *S*. *pneumoniae* showed increased survival and respiratory burst activity.^219^ In IAV followed by *Pseudomonas aeruginosa* co-infection, neutrophils showed impaired bacterial killing and this was attributed to insufficient G-CSF production.^220^ In addition, increased MMP production during IAV infection was suggested to increase lung damage during *P*. *aeruginosa* co-infection.^221^ Furthermore, blood neutrophils exposed to aspirate fluid from children with viral/bacterial co-infections showed decreased respiratory burst and killing activity against *Haemophilus influenzae* and *Staphylococcus aureus* compared to those transmigrated into the aspirate fluid from children without bacterial co-infection.^222^ Suppressive neutrophils (CD16^hi^CD62L^lo^) were also found in blood and BAL from RSV-infected infants with a bacterial co-infection.^223^

In contrast, in a mouse model of IAV-dengue virus co-infection, where both viruses were detected in the lung and mice developed pneumonia, neutrophils did not contribute to the enhanced disease.^224^ However, in a IAV-*Aspergillus fumigatus* co-infection model, mice got more severe disease and there were fewer neutrophils recruited in the superinfected mice.^225^ Overall, the data so far suggest that a viral infection inhibits the neutrophil response and render the host susceptible to a subsequent bacterial or fungal infection. Interestingly, some of our recent data suggest that a signature of activated neutrophils in the nose prior to RSV challenge correlates with the development of symptomatic infection in human volunteers.^226^ Furthermore, neutrophils recruited to the lungs of mice prior to RSV infection also increase disease severity as measured by weight loss.^226^ These findings suggest that neutrophils present in the lungs and airways, potentially attracted by a prior infection, can increase disease susceptibility and/or severity after a respiratory viral infection.

### The role of neutrophils in viral exacerbations of asthma

Viral infections commonly cause asthma exacerbations^227–229^ and infections in early life are associated with wheeze and asthma development in later life.^230^ For example, severe RSV infection is associated with later asthma development and asthma is, in turn, associated with increased susceptibility to severe RSV disease.^231^ The role of neutrophil recruitment in virus-induced exacerbations has not been fully elucidated, but it was recently shown that NETs and neutrophil enucleated cytoplasts may contribute to pathological neutrophilic inflammation in asthma.^164,232^ Furthermore, the presence of NETs and neutrophil cytoplasts in asthmatics positively correlated with higher levels of IL-17, an important mediator of neutrophil recruitment.^232^ In a recent study, it was further shown that CXCR4^hi^ neutrophils are prone to induce NETs and that these NETs increase the uptake of house dust mite by inflammatory DCs, which results in an increase in the susceptibility to allergic asthma.^162^ Also, a dysregulation of TLR7/8 signaling in neutrophils may play a role in viral-induced asthma exacerbations.^233^ For future studies it will be interesting to elucidate how neutrophils contribute to the virus-induced exacerbations in different asthma endotypes. For example, in a more neutrophil biased asthma it is possible that a virus infection, driving an increased neutrophil response, will increase disease severity.

## Summary and future research

Although neutrophils are a major effector immune cell recruited to the lungs during respiratory viral infections, their role is likely more complex than has previously been appreciated. An outstanding key question is whether neutrophils have an active role in the antiviral immune response or whether they are bystander cells recruited to the lungs and airways by virus-induced inflammation. In this review, we have summarized the current understanding of the immunological mechanisms regulating neutrophil recruitment, priming, and activation and their role during infection with different respiratory viruses. It is clear from mouse models using antibody-mediated neutrophil depletion that the effector functions of neutrophils do not have a major role in limiting viral replication or spread. However, their effect on tissue damage in the lung versus their ability to contribute to the resolution of inflammation is still unresolved and likely varies from one infection to another.

Balancing of antiviral responses in the lung is critical to manage the efficient clearance of the virus, while limiting tissue damage and avoiding compromising the lungs’ ability to perform gas exchange. In the ongoing SARS-CoV-2 pandemic as well as during severe RSV and IAV infections, dysregulated immune responses are contributing to disease severity. We therefore have to carefully consider all arms of the immune system in order to understand the underlying cause of this prolonged and heightened inflammation. The magnitude of the neutrophilic response (both in terms of the number and their activation) will determine the tissue damage that they cause. The timing of the recruitment, activation, and potentially regulation of life span in neutrophils are also key to severity of disease as both an activated neutrophilic signature prior to infection and sustained neutrophilic response are associated with disease severity in RSV, IAV, and SARS-CoV-2 infection (Fig. 1). In addition, neutrophils appear to have an underappreciated role in directing other components of both the innate and adaptive immune system. Furthermore, the presence of neutrophils might have important functions in protecting the lungs and, rather than executing a strong antiviral effect, protect the virus-infected lung from an increased exposure of commensal bacteria and fungi due to the breach in the epithelial barrier. These aspects will be crucial to study in future in vivo models.

Depleting or removing neutrophils will be a very difficult therapeutic approach as it opens up for bacterial and fungal infections to take hold and perhaps also decrease resolution of inflammation. Furthermore, it is possible that both positive and negative effects for the host response are removed in the models of disease discussed in this review and that therefore more specific knockout models, where neutrophil recruitment or effector functions are altered, will be important to fully elucidate the complex role of neutrophils during viral infections. This could also allow targeting of neutrophil function and could be a valid, future therapeutic strategy.

**Fig. 4:**
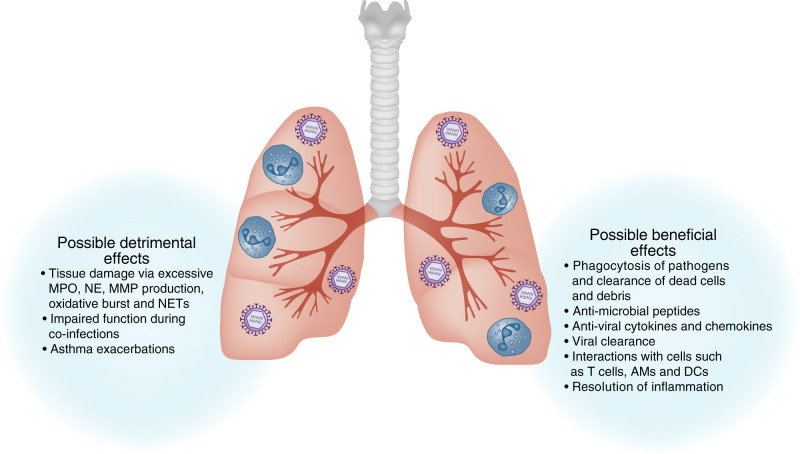
Possible beneficial and detrimental effects of neutrophils during respiratory viral infections. Neutrophils infiltrating the lungs during viral infections can aid the ongoing antiviral response by contributing to the production of antiviral cytokines and chemokines, as well as producing antimicrobial peptides. As phagocytes, neutrophils can clear pathogens and debris. These functions can aid in viral control/clearance as well as resolution of inflammation. An excess of activated neutrophils can contribute to lung tissue damage by excessive production of MPO, NE, MMPs, oxidative burst, and NETs, exacerbating disease severity.

The neutrophilic response seems to differ between types of viral infections. We suspect that neutrophils likely receive distinct signals during their recruitment, priming, and activation in the lung, which can result in the differences observed in their effector functions. Very little is known about the combination of signals, both intrinsic and extrinsic, that drive distinct neutrophil effector functions and this will be an important research avenue to pursue in the future. It is possible that we can learn from viral infections where neutrophils can have a beneficial role, to understand how to modulate neutrophil function in a positive manner during infections with RSV, IAV, and SARS-CoV-2 where neutrophils are abundantly recruited but do not appear to benefit the host. Furthermore, the transcriptional profiles and cell surface markers of possible neutrophil subtypes and their precise role in the lung of both mice and humans during respiratory infections will be important to fully understand to further answer these questions. Overall, more research into the delicate balance of beneficial and detrimental effects of neutrophils during respiratory viral infections (Fig. 4) is crucial for our understanding of the biology of these cells and our understanding of the potential possibilities, which exist to manipulate their function in targeted therapies.
